# Dental maturity and overall dental health status in children with β-thalassemia major: a case-control study

**DOI:** 10.1186/s12903-026-09457-2

**Published:** 2026-08-04

**Authors:** Muniza Omair, Sana Khurram, Ayesha Nadeem, Laibah Salman, Maryam Raza, Dua Tufail, Farzeen Manzar

**Affiliations:** 1 Department of Pathology, Dow International Dental College (DIDC), Dow University of Health Sciences, Karachi (DUHS), Karachi, Pakistan; 2 Department of Pathology and Hematology, Dow International Dental College (DIDC), Dow University of Health Sciences, Karachi, Pakistan; 3 Dow International Dental College, Dow University of Health Sciences, Karachi, Pakistan; 4 National Institute of Child Health/Sundas Foundation, Karachi, Pakistan

**Keywords:** β-thalassemia major, Delayed dental maturity, Nolla’s method, Overall dental health grade, Body mass index

## Abstract

**Objective:**

To assess dental maturity and overall dental health status in children with β-thalassemia major (β-TM) compared with healthy controls, and to examine their associations with selected clinical, anthropometric, and laboratory parameters.

**Methods:**

A hospital-based observational case-control study was conducted at Dow International Dental College (DIDC), Dow University of Health Sciences (DUHS), between March 10, 2025, and August 5, 2025. Fifty children with β-TM and fifty healthy controls aged 5–16 years were recruited using purposive sampling. Dental maturity was assessed using Nolla’s method via orthopantomograms. Periodontal and dental health was assessed using simplified periodontal screening score, the Silness–Löe Plaque Index, and tooth mobility, which were subsequently combined to generate an overall dental health grade. Data on transfusion frequency, serum ferritin, hemoglobin, and body mass index (BMI) were recorded. Analysis was performed in R software using bivariate tests, multivariable regression models adjusted for age, sex, and BMI, and age-restricted sensitivity analysis.

**Results:**

Delayed dental maturity was observed in 56% of children with β-TM and 30% of controls. Children with β-TM showed a higher simplified periodontal screening score and plaque scores than healthy controls. A poor overall dental health grade was observed in 32% of children with β-TM and 6% of healthy controls. In adjusted models, β-TM status was associated with delayed dental maturity (OR = 2.80, 95% CI 1.03–7.91, *p* = 0.046) and poorer overall dental health grade (OR = 3.17, 95% CI 1.22–8.25, *p* = 0.018). Within the β-TM group, BMI was associated with delayed dental maturity (OR = 1.12, 95% CI 1.03–1.26, *p* = 0.025). Male sex was associated with lower odds of poorer overall dental health grade (OR = 0.23, 95% CI 0.07–0.71, *p* = 0.011), while transfusion frequency was not significantly associated with dental maturity or overall dental health grade in bivariate analyses.

**Conclusion:**

Children with β-TM showed a higher frequency of delayed dental maturity and poorer overall dental health grade compared with healthy controls. BMI was associated with delayed dental maturity, whereas transfusion frequency showed no independent effect. These findings highlight the need for longitudinal studies with age-matched controls, validated dental health assessment tools, and more detailed evaluation of nutritional status, oral hygiene practices, socioeconomic factors and inflammatory markers.

**Supplementary Information:**

The online version contains supplementary material available at https://doi.org/10.1186/s12903-026-09457-2.

## Introduction

β-thalassemia major (β-TM) is an inherited blood disorder characterized by the complete or partial lack of β-globin chain production [1, 2]. This decrease or absent production causes an imbalance in the synthesis of α- and β-globin chains, leading to severe anemia, ineffective erythropoiesis, and compensatory bone marrow expansion. While blood transfusions are the main treatment approach, they inevitably cause progressive iron accumulation in vital organs such as the liver, heart, and endocrine glands. Additionally, the enlargement of the medullary space alters craniofacial structures [1]. These disruptions in systemic metabolism and craniofacial morphology significantly affect dental health and craniofacial development, ultimately affecting the quality of life [3–5].

Iron overload and the skeletal changes associated with β-TM can interfere with normal bone remodeling and dental development, leading to characteristic craniofacial abnormalities, including maxillary enlargement and malocclusion [6, 7]. These structural changes may also increase the risk of periodontal disease [6, 8, 9]. In growing children with β-TM, metabolic stress and compromised oxygen delivery impair odontogenesis and bone mineralization, which are most frequently characterized by delayed dental maturity and eruption [3, 6]. Dental maturity can be objectively assessed on radiographs using orthopantomograms (OPGs) in accordance with developmental staging systems such as Nolla’s method [3, 10].

Previous studies have reported delayed dental maturity and poorer periodontal health indicators in children with β-TM compared to healthy controls [3, 10], with clinical evaluations commonly demonstrate gingival inflammation, bleeding on probing, and increased plaque accumulation [6, 8]. Oxidative stress and iron overload are known systemic factors that may further compromise salivary gland activity and host immune responses, reducing salivary flow and local antimicrobial activity, thereby exacerbating periodontal disease [9, 11]. In addition, skeletal deformities such as malocclusion and maxillary protrusion may deter effective oral hygiene practices [4, 12].

In addition to physical and immunological alterations, clinical and hematological variables such as BMI, serum ferritin, and transfusion frequency, are considered significant factors impacting the oral health in children with β-TM [6, 13]. Furthermore, iron overload is reflected by elevated serum ferritin, has been linked to systemic oxidative stress and altered salivary environments, possibly deteriorating oral health outcomes [9, 11].

Consequently, this study aimed to assess the dental maturity and overall dental health status in children with β-TM using Nolla’s staging method and clinical indices. Additionally, these dental and oral health outcomes were correlated with selected clinical and hematological variables. The purpose of the study was to use these correlations to provide a basis for directed evidence-based dental treatment protocols in this at-risk population.

## Materials and methods

### Study design and setting

An observational case-control study evaluated dental maturity and overall dental health status among children with β-TM and healthy controls between March 10 and August 5, 2025. Children with β-TM were recruited from Sundus Foundation Blood Bank & Hematological Services Karachi, Pakistan. Oral clinical examinations and radiographic assessments were conducted at the Dow International Dental College (DIDC), Dow University of Health Sciences (DUHS), Johar campus, Karachi, Pakistan. Ethical approval of the project was obtained from the DUHS Institutional Review Board (IRB-3875/DUHS/Approval/2025/106). Written informed consent was obtained from the parent or legal guardian of each participating child before inclusion in the study.

### Sample size calculation

The sample size was calculated using the online OpenEpi sample size calculator. Based on a previously reported estimate that 92.5% of patients with β-TM may show delayed dental maturity [10], a 95% confidence level was used. We required at least 32 participants, comprising 16 children with β-TM and 16 healthy controls. A final sample of 100 was increased for greater statistical precision and to help minimize the impact of missing data or incomplete records, where 50 children were included in each group. Because this was an observational case-control study using purposive sampling, the sample size calculation was used as a minimum feasibility estimate rather than a formal power calculation for all secondary outcomes.

### Participants

A total of 100 children aged 5–16 years were recruited using purposive sampling. The study group comprised 50 children diagnosed with β-TM and 50 healthy controls without any known systemic or hematological disorders.

### Inclusion criteria

The β-TM group included male and female children aged 5–16 years with a confirmed diagnosis of β-TM based on high-performance liquid chromatography (HPLC) and/or genetic testing, who were receiving ongoing medical management. The control group consisted of healthy children who did not have any known hematological or systemic disorders.

### Exclusion criteria

Children with chronic systemic illnesses, other hematological abnormalities, or those who had undergone treatment for periodontal or dental disease during the previous six months were excluded from the study.

### Study instrument

A questionnaire (supplementary file 1) designed specifically for this study was used to gather data in order to record objective clinical, radiological, and hematological parameters. This form incorporated questions about transfusion history, iron-chelation therapy, and hematological parameters with standard assessment instruments such as Nolla’s dental maturity staging system, the Silness-Löe Plaque Index [14], and a simplified periodontal screening score for pediatric patients [15].

### Validation and testing

To ensure face and content validity, experts consisting of a pediatric dentist, a hematologist, and a biostatistician reviewed the instrument. Following this review, the questionnaire was pilot-tested on 10 children (comprising 5 children with β-TM and 5 healthy controls) to refine the phrasing and maximize clarity.

### Dental maturity assessment

Dental maturity was evaluated by applying Nolla’s method on digital orthopantomograms (OPGs). Each permanent tooth was scored from Stage 0 (no root formation) to Stage 10 (complete root closure). The scores of all teeth were summed to get a total dental maturity score, which was then converted into an estimated dental age (DA) using standard gender-specific tables.

To classify maturation, DA was compared to chronological age (CA) by calculating the age discrepancy:

$$\Delta\mathrm{A}\mathrm{g}\mathrm{e}\hspace{0.17em}=\hspace{0.17em}\mathrm{D}\mathrm{e}\mathrm{n}\mathrm{t}\mathrm{a}\mathrm{l}\:\mathrm{a}\mathrm{g}\mathrm{e}\:\left(\mathrm{D}\mathrm{A}\right)-\mathrm{C}\mathrm{h}\mathrm{r}\mathrm{o}\mathrm{n}\mathrm{o}\mathrm{l}\mathrm{o}\mathrm{g}\mathrm{i}\mathrm{c}\mathrm{a}\mathrm{l}\:\mathrm{a}\mathrm{g}\mathrm{e}\:\left(\mathrm{C}\mathrm{A}\right)$$

Participants were then classified into two groups based on this value:

- *Delayed dental maturity*: DA < CA (negative ΔAge value).
- *Normal dental maturity*: DA ≥ CA (zero or positive ΔAge value).

This identical evaluation was performed for both the β-TM group and healthy controls to compare their mean dental scores and age discrepancies.

### Periodontal examination

Oral health status was assessed using three clinical components: simplified periodontal screening score, plaque accumulation, and tooth mobility. Plaque accumulation was assessed using the Silness and Löe Plaque Index (1964) [14]. Periodontal screening was performed using a simplified Basic Periodontal Examination (BPE) approach for children and adolescents. Six index teeth were examined, including the first permanent molars and one upper and one lower incisor. The highest relevant periodontal screening score was recorded for the examined index teeth. In accordance with under-18 periodontal screening guidance, scores 0–2 were used for children aged 7–11 years, while the full score range was applied for older children and adolescents [15]. Tooth mobility was assessed according to the clinical criteria described in The Reference Manual of Pediatric Dentistry (American Academy of Pediatric Dentistry, 2024) [16]. Because dental maturity assessment was the primary outcome, children aged 5–6 years were retained in the study. Periodontal findings in this age group were recorded descriptively and interpreted cautiously.

Clinical and radiographic assessments were performed by two calibrated examiners. Before data collection, both examiners reviewed the assessment protocol and scoring criteria for Nolla staging, simplified periodontal screening score, plaque index, and tooth mobility. Prior to start the main study, interobserver reliability was assessed using 20 randomly selected radiographs and clinical records (10 from each group). Intraclass correlation coefficient (ICC) was determined for dental age scores, and Cohen’s kappa (κ) was determined for categorical indices. Reliability was excellent for dental age assessment (ICC = 0.90, 95% CI: 0.83–0.93) and substantial for Nolla staging (κ = 0.85, 95% CI: 0.80–0.89) and the periodontal indices (κ = 0.82–0.87). Three clinical components—plaque index, tooth mobility, and simplified periodontal screening score—were dichotomized for the composite score in order to facilitate a simple and comparable assessment of overall dental health status. This composite score is not a previously validated indicator; rather, it was created especially for this study to make comparing overall dental health grades easier. A score of 0 indicated no or minimal findings, while a score of 1 indicated an affected component. For the simplified periodontal screening score, score 0 was coded as 0 and scores ≥ 1 were coded as 1. For the plaque component, Silness–Löe plaque scores 0–1 were coded as 0 and scores 2–3 were coded as 1. For the mobility component, Grade 0 was coded as 0 and Grades 1–3 were coded as 1. The three binary components were summed to generate a cumulative score ranging from 0 to 3. Overall dental health grade was categorized as good for score 0, fair for score 1, and poor for scores ≥ 2.

### Simplified periodontal screening score

The simplified periodontal screening score was recorded using the six index teeth. Gentle probing was performed using a periodontal probe and mouth mirror, and the highest screening score was recorded for the examined index teeth. The simplified periodontal screening score was interpreted according to age-specific simplified BPE guidance. For children aged 7–11 years, scores were restricted to the 0–2 range, while the full score range was used for older children and adolescents. For participants under 7 years old, periodontal data were documented as clinical screening observations and evaluated conservatively. Regarding the composite overall dental health grade, a simplified periodontal screening score of 0 was coded as unaffected, whereas score ≥ 1 were coded as affected.

### Plaque index

The Silness-Löe Plaque Index [14] was used to measure plaque accumulation. Following a visual examination, the indicated index teeth were graded on a 4-point scale: 0 for no plaque, 1 for mild accumulation, 2 for moderate accumulation, and 3 for heavy accumulation. Plaque scores 0–1 were classified as unaffected for the composite overall dental health grade, while plaque scores 2–3 were classified as affected.

### Tooth mobility

Clinically, tooth mobility was assessed by exerting light, alternating pressure to opposing sides of the tooth with the rod end of a mouth mirror and the examiner’s single fingertip. Grade 0 was defined as no apparent movement, Grade 1 as horizontal movement up to 1 mm, Grade 2 as horizontal movement > 1 mm without vertical movement, and Grade 3 as significant mobility including vertical displacement [16]. For the composite overall dental health grade, Grade 0 indicated unaffected status, whereas Grades 1–3 indicated affected status. Mobility findings were interpreted cautiously, and mobility was not taken as a stand-alone indicator of periodontal disease because participants also comprised children in varying stages of dentition.

### Clinical and laboratory data

Pre-transfusion hemoglobin levels (g/dL) for each child with β-TM were taken from medical records within a week before the dental examination. Additionally, data on the type of iron chelation therapy, the frequency and length of transfusions, and treatment compliance were gathered. Serum ferritin levels (ng/mL), white blood cell (WBC) count (/µL), and platelet count (/µL) were recorded as part of hematological assessment. Nutritional status was evaluated by calculating body mass index (BMI, kg/m²) based on height (cm) and weight (kg) measurements, following World Health Organization (WHO) guidelines.

### Data handling and statistical analysis

R software version 4.5.1 was used for all statistical analysis. Depending on their distribution, continuous variables were given as means with standard deviations (SD) or medians with interquartile ranges (Q1–Q3) after being evaluated for normality. Counts and percentages were used to summarize categorical variables.

Descriptive statistics were compared between groups (β-TM vs. healthy controls) to highlight baseline differences. Continuous variables were compared using t-tests or Mann–Whitney U tests, based on data distribution. Categorical variables were analyzed using chi-square tests or Fisher’s exact tests for small sample sizes. Point-biserial correlation (r) was used for Mann–Whitney U tests, Cohen’s d was used for independent-samples t-tests, Phi coefficient was used for 2 × 2 contingency tables, and Cramer’s V was used for larger contingency tables.

Within the β-TM group, associations between clinical and dental parameters were examined using similar methods. Dental maturity was classified as delayed or normal, and overall dental health grade was categorized as good, fair, or poor based on the composite score derived from simplified periodontal screening score, plaque index, and tooth mobility. Comparisons involving three groups used one-way ANOVA or Kruskal–Wallis H tests, as appropriate based on data distribution. Effect sizes were eta squared for ANOVA and epsilon squared for Kruskal–Wallis H test.

Multivariable regression models were used to examine associations between β-TM status and dental outcomes while adjusting for age, sex, and BMI. Binary logistic regression was used for delayed dental maturity as the outcome. Ordinal logistic regression analysis was performed with overall dental health grade as the outcome variable. Multicollinearity was assessed using variance inflation factors (VIFs). The proportional-odds assumption was assessed before fitting ordinal logistic regression models. Model fit was evaluated using likelihood-ratio tests, Akaike information criterion (AIC), Bayesian information criterion (BIC), and pseudo-R² measures where applicable. Because the fitted regression models were binary logistic and ordinal logistic models, adjusted R² was not reported; pseudo-R² values were considered more appropriate for these models. Residual and influence diagnostics were performed to identify poorly fitted or highly influential observations.

Among children with β-TM, separate models were used to examine the associations of clinical and anthropometric variables with delayed dental maturity and overall dental health grade. Odds ratios (ORs) with 95% confidence intervals (CIs) were reported, and a p-value < 0.05 was considered statistically significant.

Age was considered a significant potential confounding factor since the β-TM and healthy control groups had different chronological ages. Age was included as an adjustment variable in multivariable regression models. In addition, an age-restricted sensitivity analysis was performed using the common age range of 8–16 years. This age-restricted analysis included 80 participants: 30 children with β-TM and 50 healthy controls. Age-stratified descriptive checks were also used to assess whether findings were consistent across age groups. Analyses were limited to complete cases within β-TM group to ensure data quality and consistency.

## Results

### Demographic overview and participant characteristics

The study included 100 children, of which 50 had β-TM and 50 were healthy controls. The relative proportion of males and females was comparable between the groups with 62% of the cohort being male. The median ages were 8 and 12 years for children with β-TM and the healthy controls, respectively (*p* < 0.001). The difference in age range (5–16 years) across groups was due to differences in the availability of participants; therefore, age was included as a covariate in subsequent analyses. Mean height and weight among children with β-TM (100.25 ± 22.99 cm and 21.05 ± 8.07 kg) were lower than those of healthy controls (146.9 ± 13.35 cm and 38.08 ± 9.53 kg; *p* < 0.001), suggesting altered growth patterns in the β-TM group. Median BMI was higher among children with β-TM than healthy controls (19.96 vs. 17.68 kg/m²; *p* = 0.013); however, this finding should be interpreted with caution because BMI may be influenced by short stature and altered body proportions in children with chronic hematological disease.

The median dental age was 7 years among children with β-TM and 12 years among healthy controls (*p* < 0.001). In addition, the discrepancy between dental age and chronological age was significantly greater among children with β-TM, indicating delayed dental maturity in affected children (*p* = 0.014).

### Dental maturity and overall dental health grade differences

**Fig. 1:**
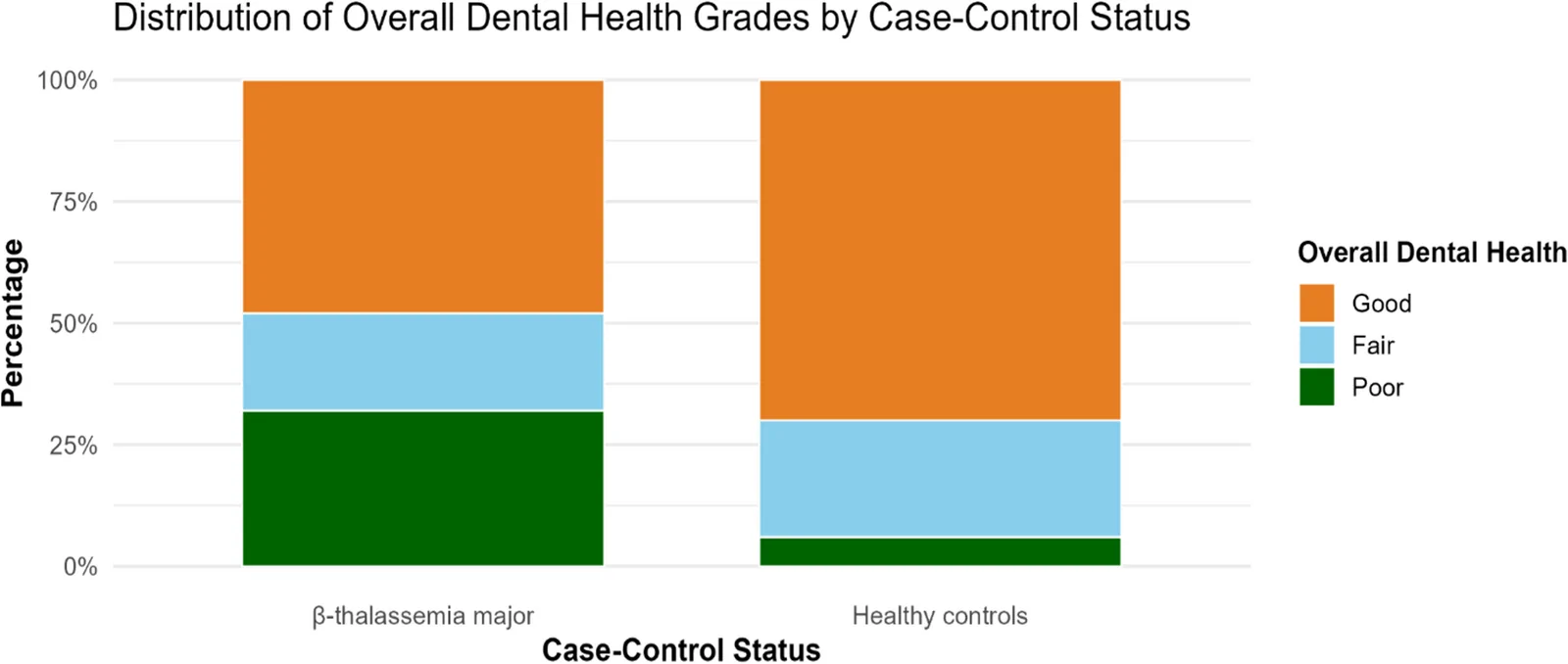
Distribution of overall dental health grade in children with β-TM (*n* = 50) and healthy controls (*n* = 50). Bars represent the percentage distribution of overall dental health grades (good, fair, poor) within each group

**Fig. 2:**
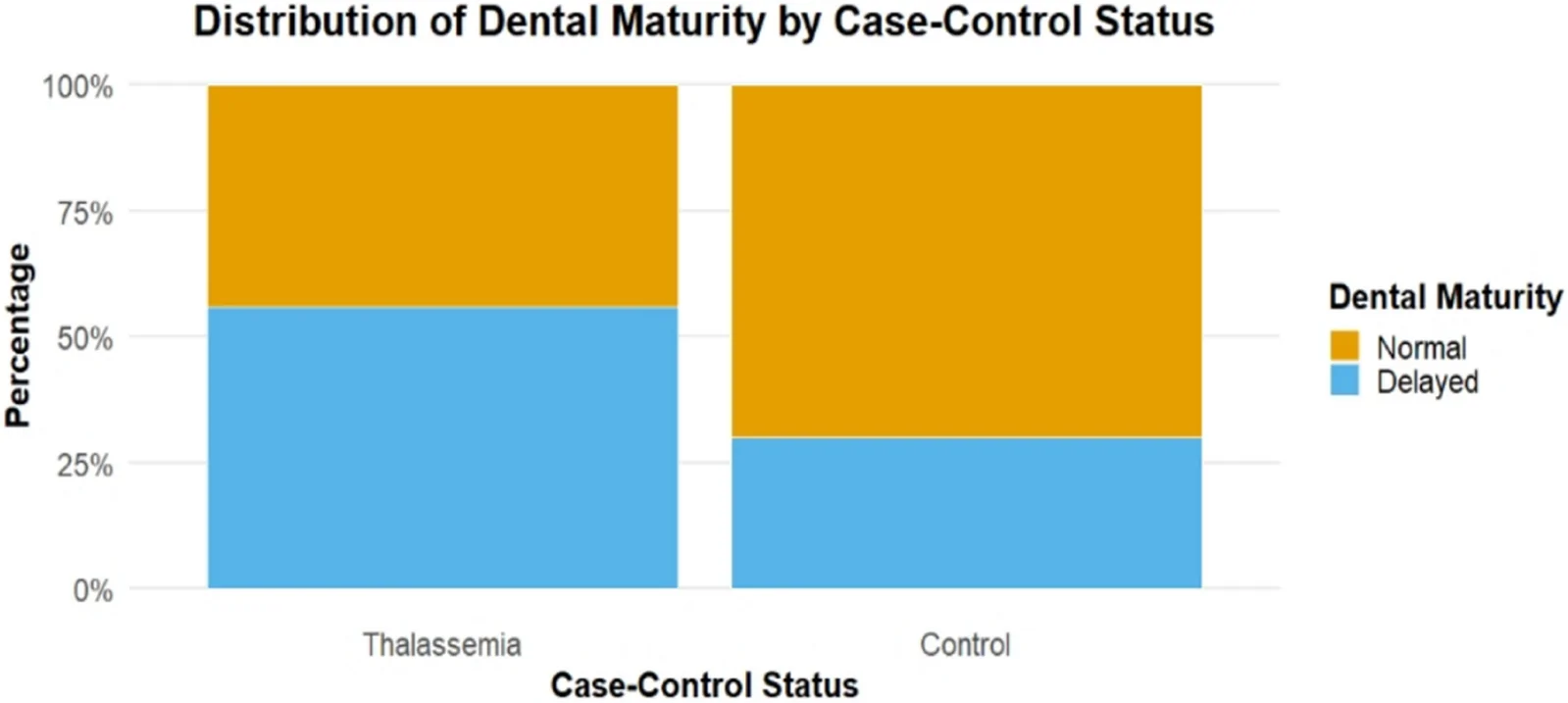
Distribution of dental maturity status (normal vs. delayed) in children with β-TM (*n* = 50) and healthy controls (*n* = 50). Bars represent the percentage distribution of dental maturity status within each group

Bivariate analyses showed that children with β-TM had a higher frequency of delayed dental maturity and poorer overall dental health grade than healthy controls. Delayed dental maturity was observed in 56% of children with β-TM compared with 30% of healthy controls (*p* = 0.015). Children with β-TM also showed higher simplified periodontal screening score and plaque index score than healthy controls (*p* = 0.0045 and *p* = 0.0156, respectively). Tooth mobility did not differ significantly between groups (*p* = 0.7254). Overall dental health grade differed between groups, with a poor overall dental health grade observed in 32% of children with β-TM compared with 6% of healthy controls (*p* = 0.0038; Table 1). The distributions of overall dental health grade and dental maturity status are shown in Figs. 1 and 2, respectively.

**Table 1:** Distributions of categorical and continuous variables, overall and stratified by β-TM status and healthy controls

Variables	Categories	Total		β-TM		Healthy controls		Effect Size	*p* value
		Frequencies/Medians/Means	Percentages/IQR/SD	Frequencies/Medians/Means	Percentages/IQR/SD	Frequencies/Medians/Means	Percentages/IQR/SD		
Gender ^a^	Male	62	62.00%	31	62.00%	31	62%	0.00	1.000
	Female	38	38.00%	19	38.00%	19	38%	0.00	1.000
Dental Maturity Status ^a^	Normal	57	57.00%	22	44.00%	35	70%	0.24	0.0154
	Delayed	43	43.00%	28	56.00%	15	30%		
Simplified Periodontal Screening Score^a^	Score 0	77	77.0%	31	62.0%	46	92%	0.36	0.0045
	Score 1	14	14.0%	12	24.0%	2	4%		
	Score 2	8	8.0%	6	12.0%	2	4%		
	Score 3	0	0.0%	0	0.0%	0	0.0%		
	Score 4	1	1.0%	1	2.0%	0	0.0%		
Plaque Index ^a^	Grade 0	24	24.0%	7	14.0%	17	34%	0.32	0.0156
	Grade 1	53	53.0%	26	52.0%	27	54%		
	Grade 2	20	20.0%	14	28.0%	6	12%		
	Grade 3	3	3.0%	3	6.0%	0	0.0%		
Tooth Mobility ^a^	Grade 0	79	79.0%	38	76.0%	41	82%	0.11	0.7254
	Grade 1	11	11.0%	6	12.0%	5	10%		
	Grade 2	9	9.0%	5	10.0%	4	8%		
	Grade 3	1	1.0%	1	2.0%	0	0.0%		
Overall Dental Health Grade ^a^	Good	59	59.0%	24	48.0%	35	70.0%	0.33	0.0038
	Fair	22	22.0%	10	20.0%	12	24.0%		
	Poor	19	19.0%	16	32.0%	3	6.0%		
Age(years) ^b^		11	[8–13]	8	[6.25-12]	12	[11–14]	-0.54	< 0.001
Height(cm) ^c^		123.58	29.99	100.25	22.99	146.9	13.35	-2.48	< 0.001
Weight(kg) ^c^		29.56	12.27	21.05	8.07	38.08	9.53	-1.93	< 0.001
Body Mass Index(kg/m²) ^b^		18.26	[15.52–21.74]	19.96	[15.44–26.21]	17.68	[15.95–19.14]	0.29	0.0129
Dental age(years) ^b^		11	[7–13]	7	[6–11]	12	[11-13.75]	-0.63	< 0.001
Dental age -chronological age difference (years) ^b^		0	[-1–0.125]	-0.75	[-1.375–0]	0	[-1–1]	-0.28	0.014

*Abbreviations*: *β-TM *β-thalassemia major, *BMI *body mass index, *SD *standard deviation, *IQR *interquartile range

ᵃ Categorical variables are presented as frequency (%). The chi-square test or Fisher’s exact test was used, as appropriate. Effect size is reported as Phi (φ) for 2 × 2 tables and Cramer’s V for larger tables

ᵇ Non-normally distributed continuous variables are presented as median [Q1–Q3]. The Mann–Whitney U test was used, with rank-biserial correlation reported as the effect size

ᶜ Normally distributed continuous variables are presented as mean ± SD. The independent-samples t-test was used, with Cohen’s d reported as the effect size

### Multivariable associations with dental outcomes

In multivariable models adjusted for age, sex, and BMI, β-TM status was associated with higher odds of delayed dental maturity (OR = 2.80, 95% CI 1.03–7.91, *p* = 0.046). β-TM status was also associated with poorer overall dental health grade (OR = 3.17, 95% CI 1.22–8.25, *p* = 0.018). Age, sex, and BMI were not independently associated with delayed dental maturity or overall dental health grade in the adjusted full-sample models. Model diagnostics did not identify major concerns affecting model interpretation. Multicollinearity was low, with all variance inflation factor (VIF) values below 1.5. The proportional-odds assumption for the ordinal logistic regression model was satisfied (*p* = 0.925). Review of residual and influence diagnostics did not identify observations requiring exclusion; therefore, all complete cases were retained in the final analyses. Model fit indicators, including likelihood-ratio tests, AIC, BIC, and pseudo-R² values, are presented in Supplementary Table S3.

### Clinical and hematological characteristics of children with β-TM

Among children with β-TM, most were diagnosed via HPLC (88%) and presented with severe anemia (92%). More than half of the children were diagnosed within the first year of life, and nearly half required transfusions every 15–16 days. Endocrinopathies (26%) were the most common comorbidity, and the majority (90%) were receiving iron chelation therapy. Hematological assessment confirmed severe anemia (mean hemoglobin 5.72 ± 1.65 g/dL) and markedly elevated ferritin levels (median 2992 ng/mL; Table 2).

**Table 2:** Clinical, treatment-related, and hematological characteristics of children with β-TM (*n* = 50)

Variables ^a^	Categories	Frequencies/Means/Medians	Percentages/SD/IQR
Diagnosis	HPLC	44	88.00%
	Gene Mutation	6	12.00%
Anemia Stage	Moderate	4	8.00%
	Severe	46	92.00%
Time of Diagnosis of Thalassemia	At Birth	4	8.00%
	0–12 months	27	54.00%
	Between Age 1–5	16	32.00%
	After 5 years	3	6.00%
Comorbidities	None	31	62.00%
	Cardiovascular Disorders	3	6.00%
	Endocrinopathy	13	26.00%
	Diabetes Mellitus	1	2.00%
	Epilepsy	1	2.00%
	Hepatitis C Virus	1	2.00%
Frequency of Blood Transfusion	Every 15–16 days	24	48.00%
	Once a month	21	42.00%
	After more than a month	5	10.00%
Age of Primary Tooth Loss	Under 5 years	4	8.00%
	5–6 years	8	16.00%
	Above 6 years	9	18.00%
	Not Yet	7	14.00%
	Don’t Remember	22	44.00%
Use of Iron Chelating Medications	No	5	10.00%
	Yes	45	90.00%
Hemoglobin (g/dL) ^b^		5.72	1.65
WBCs (/µL) ^c^		7600	[6000–8500]
Platelets (/µL) ^b^		224,315	105621.74
Ferritin (ng/mL) ^c^		2992	[2303–3751]

*Abbreviations*: *HPLC* high-performance liquid chromatography, *WBC* white blood cell, *SD* standard deviation, *IQR* interquartile range

^b^ Frequencies and percentages were reported

^b^ Means and SD were reported

^c^ Medians and IQR were reported

### Predictors of delayed dental maturity among children with β-TM

**Fig. 3:**
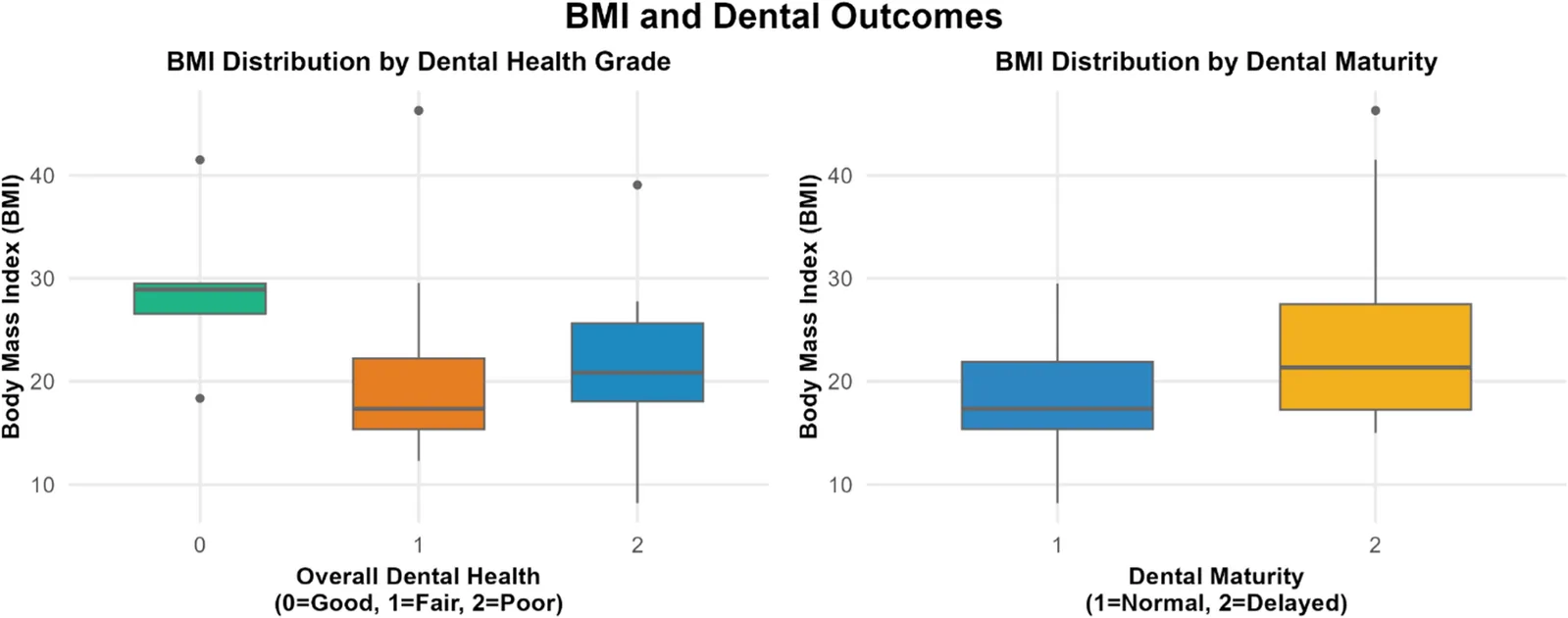
Boxplots of BMI by dental maturity status and overall dental health grade among children with β-TM. The left panel shows BMI distribution by overall dental health grade (0 = good, 1 = fair, 2 = poor), and the right panel shows BMI distribution by dental maturity status (1 = Normal, 2 = Delayed)

Within β-TM group, categorical predictors such as anemia severity, transfusion frequency, and comorbidities were not associated with dental maturity (Table 3). Among continuous variables, only BMI showed a significant association, with higher BMI linked to delayed dental maturity (*p* = 0.035; Table 4). Boxplots of BMI (kg/m²) by overall dental health grade and dental maturity status are shown in Fig. 3. In multivariable logistic regression adjusted for age, BMI remained associated with delayed dental maturity within the β-TM group (OR = 1.12, 95% CI 1.03–1.26, *p* = 0.025; Table 5). These findings suggest an association between BMI and delayed dental maturity within the β-TM group. However, BMI should be interpreted cautiously in this population because altered growth patterns and short stature may affect BMI interpretation.

**Table 3:** Association of categorical predictors with dental maturity status (normal vs. delayed) and overall dental health grade (good, fair and poor) among children with β-TM

Variable ^a^	Dental Maturity		Overall Dental Health Grade	
	Effect Size	*p* value	Effect Size	*p* value
Gender	0.01	0.934508	0.55	0.0005
Anemia Group ^b^	-0.11	0.620851	0.26	0.1872
Time of Diagnosis of Thalassemia	0.22	0.481901	0.32	0.1121
Frequency of Blood Transfusion	0.27	0.296909	0.17	0.5649
Age of Primary Tooth Loss	0.30	0.472979	0.32	0.4689
Iron Chelating Medications Usage ^b^	0.11	0.642831	0.17	0.4879
Comorbidities Groups	0.28	0.283547	0.20	0.6868

Effect size is reported as phi coefficient for 2 × 2 tables and Cramer’s V for larger tables

*Abbreviations*: *β-TM* β-thalassemia major

^a^ Chi square test was applied for all variables

^b^ Fisher’s exact test was applied for anemia group and iron chelating medications usage for comparison with dental maturity

**Table 4:** Comparison of continuous variables between patients with normal and delayed dental maturity

Variable	Normal	Delayed Maturity	Effect Size	*p* value
Age (years) ^a^	7 [6–11.75]	8 [7–12]	-0.15	0.3603
BMI (kg/m²) ^a^	17.36 [15.38–21.92]	21.35 [17.26–27.49]	-0.35	0.0348
Dental Age (years) ^a^	7.5 [6.5-12.75]	7 [6–11]	0.25	0.1352
Hemoglobin (g/dL) ^b^	5.82 ± 1.53	5.64 ± 1.77	0.11	0.6974
WBCs (/µL) ^a^	7000 [6100–8100]	7900 [6000–9300]	-0.19	0.3135
Platelets (/µL) ^b^	215105.26 ± 111456.47	233526.32 ± 101636.81	-0.17	0.5978
Ferritin (ng/mL) ^a^	2994 [2687–3602]	2940 [2087.2–4052]	0	1

*Abbreviations*: *BMI* body mass index, *WBC* white blood cell, *SD* standard deviation, *IQR* interquartile range

^a^ Non-normal variables. Medians (Q1-Q3) and IQR were reported. Mann-Whitney U test was applied

^b^ Normally distributed variables are presented as mean ± SD. Independent-samples t-test was used

**Table 5:** Multivariable regression models examining associations with delayed dental maturity and overall dental health grade

Outcome ^a^	Predictor	OR	95% CI lower	95% CI upper	*p* value
Delayed dental maturity	β-TM status	2.80	1.03	7.91	0.046
Delayed dental maturity	Age	1.03	0.89	1.21	0.663
Delayed dental maturity	Male sex	0.74	0.31	1.76	0.504
Delayed dental maturity	BMI	1.06	0.99	1.15	0.100
Overall dental health grade	β-TM status	3.17	1.22	8.25	0.018
Overall dental health grade	Age	0.97	0.84	1.12	0.672
Overall dental health grade	Male sex	0.56	0.25	1.28	0.170
Overall dental health grade	BMI	1.00	0.94	1.07	0.980

Model fit was assessed using likelihood-ratio testing, AIC, BIC, and pseudo-R² measures. Conventional adjusted R² values were not reported because binary logistic and ordinal logistic regression models were used; pseudo-R² measures were considered more appropriate for these regression models

*Abbreviations*: *β-TM* β-thalassemia major, *OR* odds ratio, *CI* confidence interval, *BMI* body mass index

^a^ Binary logistic regression was used for delayed dental maturity. Ordinal logistic regression was used for overall dental health grade. Models were adjusted for age, sex, and BMI. β-TM status was coded as case versus control

### Predictors of overall dental health grade among children with β-TM

**Fig. 4:**
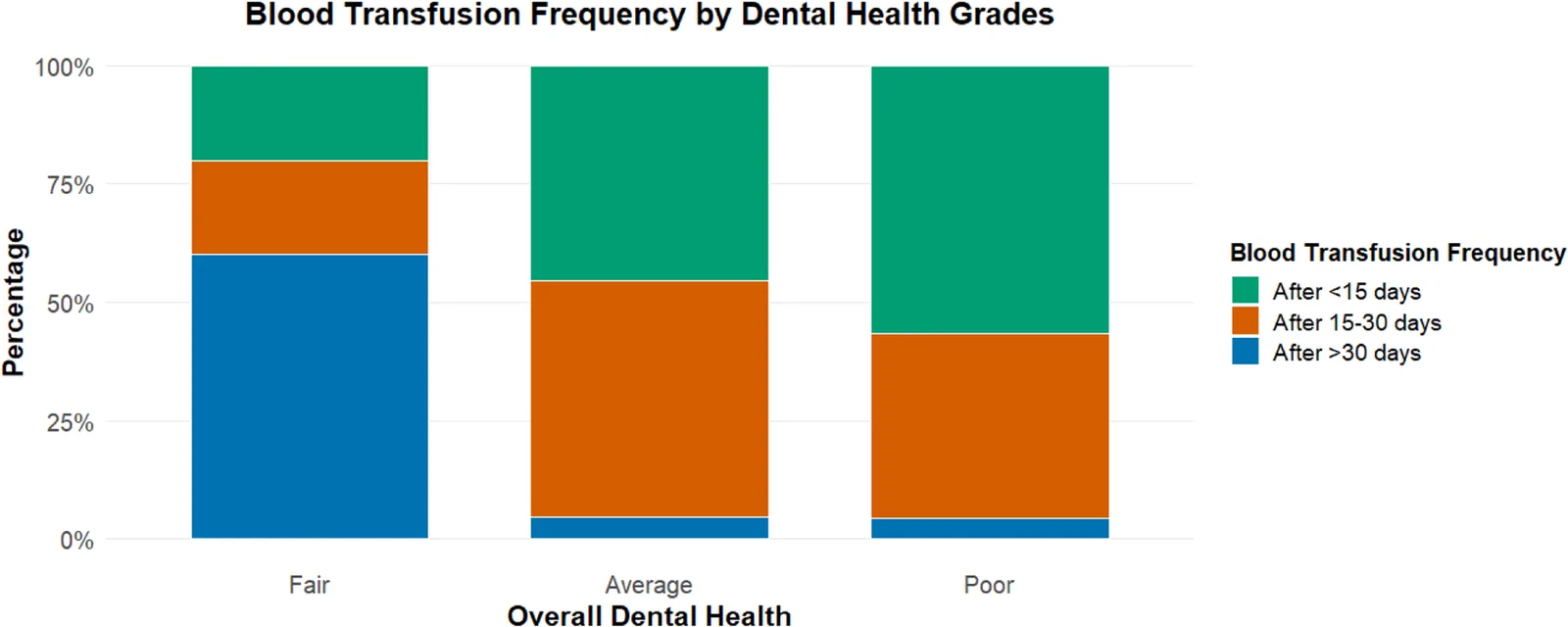
Distribution of blood transfusion frequency across overall dental health grades among children with β-TM

Analysis of overall dental health grade among children with β-TM showed a significant association with sex in bivariate analysis (Table 3). Poorer overall dental health grades were more frequent among female participants, while male sex was associated with lower odds of poorer overall dental health grade in the ordinal regression model (OR = 0.23, 95% CI 0.07–0.71, *p* = 0.011; Table 5). Transfusion frequency was not significantly associated with overall dental health grade in the updated bivariate analysis or in the adjusted ordinal regression model. Among continuous variables, hemoglobin differed across overall dental health grades, whereas BMI, platelet count, and ferritin were not significantly associated with overall dental health grade in the updated analysis (Table 6). These findings should be interpreted cautiously because of the modest subgroup size. The distribution of transfusion frequency across overall dental health grade is shown descriptively in Fig. 4.

**Table 6:** Comparison of continuous variables across overall dental health grades among children with β-TM

Variable	Good	Fair	Poor	Effect Size	*p* value
Age (years) ^a^	8.00 [6.00–13.00]	9.00 [7.00–10.00]	8.00 [7.00–11.00]	-0.04	0.8437
BMI (kg/m²) ^a^	18.43 [15.36–26.37]	18.95 [15.36–24.25]	21.10 [18.77–24.75]	-0.02	0.5787
Dental Age ^a^	7.00 [5.75–11.12]	7.50 [6.62–8.88]	7.50 [6.38–11.12]	-0.03	0.8248
Hemoglobin (g/dL) ^a^	5.60 [4.88–7.05]	7.00 [6.30–7.78]	4.70 [4.20–5.58]	0.10	0.0370
WBCs (/µL) ^a^	7600.00 [5750.00–10425.00]	8000.00 [7725.00–9175.00]	7700.00 [5500.00–9050.00]	-0.02	0.5733
Platelets (/µL) ^b^	296166.67 ± 223625.01	261700.00 ± 120947.23	205187.50 ± 129361.62	0.05	0.3048
Ferritin (ng/mL) ^a^	2898.00 [2029.00–4146.50]	3271.00 [2910.00–3619.25]	3081.00 [2439.00–3658.50]	-0.05	0.8577
Dental Age–Chronological Age Difference (years) ^a^	-1.00 [-1.12–0.00]	-0.25 [-1.38–0.00]	-0.75 [-1.12–0.25]	-0.04	0.9520

*Abbreviations*: *BMI* body mass index, *WBC* white blood cell, *SD* standard deviation, *IQR* interquartile range

^a^ Non-normal variables are presented as median [Q1–Q3]. Kruskal–Wallis H test was used

^b^ Normally distributed variables are presented as mean ± SD. One-way ANOVA was used

In the age-restricted sensitivity analysis including participants aged 8–16 years (*n* = 80; 30 children with β-TM and 50 healthy controls), β-TM status remained associated with delayed dental maturity after adjustment for age, sex, and BMI (OR = 4.63, 95% CI 1.57–14.83, *p* = 0.007). The association with overall dental health grade was attenuated in the adjusted sensitivity model (OR = 2.58, 95% CI 0.93–7.20, *p* = 0.070). The age-restricted sensitivity model and descriptive age-stratified checks are presented in Supplementary file 2 (Tables S1 and S2). These sensitivity findings were interpreted cautiously because the age-restricted sample reduced the number of β-TM and some outcome categories contained small numbers.

## Discussion

This case-control study evaluated dental maturity and overall dental health grade in children with β-TM compared with healthy controls. Children with β-TM showed a poorer overall dental health grade and increased frequency of delayed dental maturity. In multivariable models, after controlling for age, sex, and BMI, these associations remained significant. However, these results should be considered as associations rather than causal inferences because of the observational case-control study design.

The higher frequency of delayed dental maturity in the present study corresponds with the observations of Abdel Hakeem et al. (3), who reported similar findings in children with β-TM. The underlying mechanism is probably multifactorial, with chronic anemia, tissue hypoxia, iron overload, and altered bone remodeling contributing to delayed dental maturity.

The cellular and metabolic processes required for dental maturity and eruption may be disrupted by chronic anemia through reduced tissue oxygenation. Moreover, repeated blood transfusions invariably result in iron overload, which produces reactive oxygen species that may change the mineralization processes necessary for dental maturity [1, 7, 17]. Chronic anemia also causes compensatory bone marrow hyperplasia, which modifies alveolar and craniofacial growth patterns [5, 12]. Despite the biological plausibility of these pathways, this study found no statistically significant association between hemoglobin or serum ferritin levels and delayed dental maturity. This lack of association suggests that standard hematological indices fail to mirror the complex biological pathways influencing dental maturity. Consequently, dental maturity appears to be driven by an intricate interplay of systemic, developmental, and environmental variables. These multifaceted interactions are likely too complex to be captured by conventional blood indices alone.

Children with β-TM had poorer overall dental health grades than healthy controls. This disparity was reflected by higher simplified periodontal screening scores and increased plaque accumulation. The composite dental health grade, based on the simplified periodontal screening score, plaque index, and tooth mobility, reflected poorer overall dental health grade in these children. Similar findings have been reported previously, with studies describing increased gingival inflammation and poorer oral hygiene practices among children with β-TM [8, 9].

β-TM is likely associated with poorer oral health through mechanisms such as chronic anemia, iron overload, oxidative stress, and immune dysregulation. These findings should be interpreted with caution, as several potentially important confounding factors were not assessed in the present study. Socioeconomic status can affect access to preventive dental care, utilization of dental services, and oral hygiene practices, which may influence plaque accumulation and periodontal health. Similarly, dietary behaviors and oral hygiene practices may independently alter oral health status and contribute to the differences observed between children with β-TM and healthy controls. Furthermore, periodontal tissues, salivary function, bone metabolism, and oral maturity may be impacted by chronic inflammatory burden and nutritional disorders linked to β-TM through pathways not fully reflected by standard hematological indicators [9, 11, 13, 18]. As a result, variations in dental maturity and overall dental health grade may be the result of a mix of biological impacts associated with the disease and unmeasured environmental, behavioral, and nutritional factors. These findings suggest that dental maturity and overall dental health may be more significantly affected by the long-term biological effect of β-TM than transfusion frequency, which serves as a proxy for overall treatment intensity. To better differentiate β-TM-specific effects from potentially modifiable risk factors, future prospective studies incorporating socioeconomic indicators, detailed oral hygiene assessments, dietary evaluation, inflammatory biomarkers, salivary parameters, and comprehensive nutritional assessment are required.

The association of BMI with dental maturity and overall dental health outcomes in chronic hematological disorders is complex. The reduced BMI and impaired physical growth in children with β-TM have been reported in some studies, which was usually attributed to chronic anemia, malnutrition and endocrine dysfunction [13, 19]. Conversely, our case-control data showed that BMI was higher among children with β-TM than among healthy controls, thus this should be interpreted cautiously. Although BMI is widely used to assess nutritional status, it may be poorly representative in patients with β-TM due to confounding variables including short stature, alterations in body proportions and underlying growth deficits. Previous research has indicated that BMI is a poor marker of nutrition in transfusion dependent β-TM [20]. An association was present between delayed dental maturity and BMI in our β-TM cohort, but no association with overall dental health grade, suggesting outcome-specific effects.

In summary, the differences in dental outcomes which have been observed are likely to be a result of a multifactorial interaction between systemic factors and local oral environmental conditions. Chronic anemia, tissue hypoxia, bone marrow expansion, iron overload and oxidative stress appear to synergize in causing abnormal craniofacial development, delayed odontogenesis and impaired periodontal health [3, 13, 17]. Nevertheless, in this case-control study important mechanistic variables including biomarkers of oxidative stress, salivary flow and composition, oral hygiene behavior or detailed nutritional status measures were not evaluated. Thus, these mechanisms cannot be ruled out but remain to be confirmed by additional biological studies.

The present findings also have potential clinical implications. Children with β-TM may benefit from routine oral health surveillance integrated into their multidisciplinary care. Early dental assessments, regular plaque control, preventive periodontal screening, and caregiver-directed oral hygiene education may facilitate earlier identification of delayed dental maturity and periodontal problems. Close collaboration between pediatric dentists, hematologists, pediatricians, and nutrition specialists could support comprehensive management by addressing both systemic disease and modifiable oral health risk factors.

This study has several limitations. First, controls were not individually age-matched to cases, which may have introduced residual confounding despite statistical adjustment. Therefore, age-adjusted models and age-restricted sensitivity analysis were performed, but residual confounding by age cannot be excluded. Furthermore, many potentially important confounding variables were not evaluated, such as socioeconomic status, detailed oral hygiene practices, dietary intake, and comprehensive nutritional assessment. Since these factors may influence oral health outcomes independently, their inclusion in future studies would provide a more comprehensive assessment. Second, this was an observational case-control study using purposive sampling, the sample size calculation was used as a minimum feasibility estimate rather than a formal power calculation for all secondary outcomes. As a result, secondary analyses should therefore be interpreted with caution and validated in large prospective studies. Although regression diagnostics did not identify major concerns affecting model interpretation, the relatively modest sample size limited the precision of some estimates and reduced the ability to detect influential observations with high confidence. Third, BMI was used as a practical anthropometric measure, but BMI may not fully reflect nutritional or growth status in children with β-TM. Fourth, tooth mobility findings in mixed dentition should be interpreted cautiously because physiological exfoliation-related mobility may occur. Fifth, some regression estimates had wide confidence intervals, reflecting the modest sample size and subgroup distributions. Finally, because the study was case-control, findings should be interpreted as associations rather than causal relationships.

## Conclusion

Children with β-TM displayed a higher frequency of delayed dental maturity and overall lower dental health grades compared to the healthy controls. Full sample model demonstrated that these associations remained significant after adjusting for age, sex and BMI. In the age restricted sensitivity analysis, the association with delayed dental maturity persisted, whereas attenuated with overall dental health grade. The frequency of transfusion was not significantly associated with either outcome, indicating the differential dental findings likely reflect the cumulative and multifactorial burden of β-TM, rather than treatment intensity. Regular dental examinations, periodontal care and teaching proper oral hygiene should be part of these children’s multidisciplinary management. However, these findings should be interpreted with caution due to the case-control design, modest sample size, baseline differences in age and the potential influence of unmeasured nutritional, behavioral and socioeconomic factors. Although age-adjusted regression models and age-restricted sensitivity analyses produced largely consistent findings, residual confounding by age cannot be completely excluded. Larger prospective studies using age-matched controls and a thorough analysis of these potential risk factors are required to confirm the findings and clarify the mechanisms affecting dental maturity and overall dental health in children with β-TM.

## Supplementary Information

Supplementary Material 1.

Supplementary Material 2.

## Data Availability

The datasets used and/or analyzed during the current study are available from the corresponding author on reasonable request.

## Abbreviations

β-TM: β-Thalassemia Major
Hb: Hemoglobin
PI: Plaque Index
BPE: Basic periodontal examination
OPG: Orthopantomogram
HPLC: High Performance Liquid Chromatography
R: R statistical Software
