# Supplementary material for: Dental maturity and overall dental health status in children with β-thalassemia major: a case-control study
Source: BMC Oral Health. 2026 Aug 4;26:2168. doi: 10.1186/s12903-026-09457-2 (PMC13729425; doi:10.1186/s12903-026-09457-2)
Supplement: Supplementary file 1 [file 12903_2026_9457_MOESM1_ESM.docx]

**Supplementary File 1: Study Proforma**

**Dental Development and Periodontal Status in Children with β-Thalassemia Major: A Case-Control Study**

### Section A: Participant Identification

- **Participant ID:** ______
- **Group:** 1 **=** Case (β-TM), 2 = Control
- **Date of Examination:** ___ / ___ / ____

### Section B: Demographic Data

- **Age (years):** ______
- **Gender:** 1 = Male, 2 = Female
- **Height (cm):** ______
- **Weight (kg):** ______
- **BMI (kg/m²):** ______

### Section C: Medical History (Cases Only)

- **Diagnosis confirmation:** 1 = HPLC, 2 = Genetic testing
- **Age at diagnosis (years):** ______
- **Transfusion frequency:** 1 = Every 15–16 days, 2 = Once a month, 3 = After more than a month
- **Duration of transfusion (years):** ______
- **Iron chelation therapy:** 0 = No, 1 = Yes
- **Type of chelation:** 1 = Deferasirox, 2 = Deferoxamine, 3 = Other
- **Adherence to therapy:** 1 = Good, 2 = Irregular, 3 = Poor

### Section D: Laboratory Parameters

- **Pre-transfusion Hb (g/dL):** ______
- **Serum Ferritin (ng/mL):** ______
- **WBC count (/µL): ______**
- **Platelet count (/µL): ______**

### Section E: Dental and Medical Assessment

**Medical and Family History:** -

Co-morbid conditions (if any): ☐ Hypertension ☐ Diabetes Mellitus ☐ Cardiovascular Disease ☐ Other: ______

Blood transfusion frequency: ☐ Every 15–16 days ☐ Once a month ☐ After more than a month

**Dental History:** -

OPG (Orthopantomogram) done? ☐ Yes ☐ No
If yes, Year: ______

History of periodontal diseases? ☐ Yes ☐ No

Age of first milk tooth loss: ☐ Under 5 ☐ 5–6 ☐ 6 or more ☐ Don’t remember

Iron profile tests done? ☐ Yes ☐ No
If yes, Date of sample: ______
Ferritin: ______
Pre-transfusion Hb: ______

### Section F: Dental Maturity Assessment (OPG-Based)

- **OPG performed:** 0 = No, 1 = Yes
- **Nolla staging for each assessable permanent tooth (0–10):** Tooth No.: ______, Nolla Stage: ______
- **Mean Nolla Score:** ______
- **Dental Age (years):** ______
- **Chronological Age (years):** ______
- **Dental Maturity Status:** 0 = Normal, 1 = Delayed

**Delayed dental maturity was recorded when dental age was lower than chronological age.**

| 17 | 16 | 15 | 14 | 13 | 12 | 11 | 21 | 22 | 23 | 24 | 25 | 26 | 27 |
| --- | --- | --- | --- | --- | --- | --- | --- | --- | --- | --- | --- | --- | --- |
|  |  |  |  |  |  |  |  |  |  |  |  |  |  |
|  |  | 55 | 54 | 53 | 52 | 51 | 61 | 62 | 63 | 64 | 65 |  |  |
|  |  |  |  |  |  |  |  |  |  |  |  |  |  |
|  |  | 85 | 84 | 83 | 82 | 81 | 71 | 72 | 73 | 74 | 75 |  |  |
|  |  |  |  |  |  |  |  |  |  |  |  |  |  |
| 47 | 46 | 45 | 44 | 43 | 42 | 41 | 31 | 32 | 33 | 34 | 35 | 36 | 37 |

### Section G: Periodontal Examination

1. **Plaque Index (Silness & Löe)** - Score 0–3, Binary conversion: 0–1 = 0, 2–3 = 1 - Final Plaque Score: ______

| 17 | 16 | 15 | 14 | 13 | 12 | 11 | 21 | 22 | 23 | 24 | 25 | 26 | 27 |
| --- | --- | --- | --- | --- | --- | --- | --- | --- | --- | --- | --- | --- | --- |
|  |  |  |  |  |  |  |  |  |  |  |  |  |  |
|  |  | 55 | 54 | 53 | 52 | 51 | 61 | 62 | 63 | 64 | 65 |  |  |
|  |  |  |  |  |  |  |  |  |  |  |  |  |  |
|  |  | 85 | 84 | 83 | 82 | 81 | 71 | 72 | 73 | 74 | 75 |  |  |
|  |  |  |  |  |  |  |  |  |  |  |  |  |  |
| 47 | 46 | 45 | 44 | 43 | 42 | 41 | 31 | 32 | 33 | 34 | 35 | 36 | 37 |

2. **Simplified Periodontal Screening Score -** Record the highest periodontal screening score for the six index teeth: UR6, UR1, UL6, LL6, LL1, and LR6. For children aged 7–11 years, use scores 0–2. For older children/adolescents, use the full score range. For children younger than 7 years, record findings as clinical screening observations and interpret cautiously. Raw screening score: ______ Composite component score: 0 = screening score 0; 1 = screening score ≥1

| 17 | 16 | 15 | 14 | 13 | 12 | 11 | 21 | 22 | 23 | 24 | 25 | 26 | 27 |
| --- | --- | --- | --- | --- | --- | --- | --- | --- | --- | --- | --- | --- | --- |
|  |  |  |  |  |  |  |  |  |  |  |  |  |  |
|  |  | 55 | 54 | 53 | 52 | 51 | 61 | 62 | 63 | 64 | 65 |  |  |
|  |  |  |  |  |  |  |  |  |  |  |  |  |  |
|  |  | 85 | 84 | 83 | 82 | 81 | 71 | 72 | 73 | 74 | 75 |  |  |
|  |  |  |  |  |  |  |  |  |  |  |  |  |  |
| 47 | 46 | 45 | 44 | 43 | 42 | 41 | 31 | 32 | 33 | 34 | 35 | 36 | 37 |

3. **Tooth Mobility** - Grade 0 = No mobility, Grade 1 ≤1 mm horizontal, Grade 2 >1 mm horizontal, Grade 3 Vertical mobility - Binary conversion: Grade 0 = 0, Grades 1–3 = 1 - Final Mobility Score: ______

| 17 | 16 | 15 | 14 | 13 | 12 | 11 | 21 | 22 | 23 | 24 | 25 | 26 | 27 |
| --- | --- | --- | --- | --- | --- | --- | --- | --- | --- | --- | --- | --- | --- |
|  |  |  |  |  |  |  |  |  |  |  |  |  |  |
|  |  | 55 | 54 | 53 | 52 | 51 | 61 | 62 | 63 | 64 | 65 |  |  |
|  |  |  |  |  |  |  |  |  |  |  |  |  |  |
|  |  | 85 | 84 | 83 | 82 | 81 | 71 | 72 | 73 | 74 | 75 |  |  |
|  |  |  |  |  |  |  |  |  |  |  |  |  |  |
| 47 | 46 | 45 | 44 | 43 | 42 | 41 | 31 | 32 | 33 | 34 | 35 | 36 | 37 |

### **Section H: Overall Dental Health Grade**

- **Plaque component score:** 0/1
- **Simplified periodontal screening component score:** 0/1
- **Mobility component score:** 0/1
- **Total composite score (0–3): ______**
- Overall dental health grade: Good = total score 0; Fair = total score 1; Poor = total score 2–3

### Section I: Lifestyle and Dietary Habits

- Use of iron-chelating medication? ☐ Yes ☐ No
- Dietary restrictions related to thalassemia? ☐ Yes ☐ No
  If yes, specify: ______
- Symptoms: ☐ Gum bleeding ☐ Gum swelling ☐ Bad breath ☐ Loose teeth ☐ Tooth sensitivity

### Section J: Notes / Remarks

- Additional observations/comments: ______
- Dental maturity score reference: Nolla stages of tooth calcification/development on OPG (0–10).

**Instructions for Data Collection:** - Fill in participant-specific information in the spaces provided. - Use the coded entries for uniformity. - Record clinical parameters using standardized techniques and mark observations clearly. - Repeat measurements where required to ensure accuracy.
