# Supplementary material for: Dental maturity and overall dental health status in children with β-thalassemia major: a case-control study
Source: BMC Oral Health. 2026 Aug 4;26:2168. doi: 10.1186/s12903-026-09457-2 (PMC13729425; doi:10.1186/s12903-026-09457-2)
Supplement: Supplementary file 2 [file 12903_2026_9457_MOESM2_ESM.docx]

**Table S1. Sensitivity Analysis: Multivariable Regression Models Restricted to Common Age Range (8–16 years)**

| **Outcome** | **Predictor** | **OR** | **95% CI** | **p-value** |
| --- | --- | --- | --- | --- |
| **Delayed Dental Maturity** | β-thalassemia major status | 4.63 | 1.57–14.83 | 0.007 |
|  | Age | 0.86 | 0.69–1.06 | 0.156 |
|  | Male sex | 0.73 | 0.26–2.00 | 0.553 |
|  | Body Mass Index | 1.00 | 0.90–1.12 | 0.971 |
| **Overall Dental Health Grade** | β-thalassemia major status | 2.58 | 0.93–7.20 | 0.070 |
|  | Age | 0.87 | 0.71–1.06 | 0.162 |
|  | Male sex | 0.84 | 0.32–2.16 | 0.712 |
|  | Body Mass Index | 1.07 | 0.98–1.18 | 0.147 |

Models were restricted to participants aged 8–16 years (n = 80; 30 β-thalassemia major cases and 50 healthy controls). Binary logistic regression was used for delayed dental maturity, and ordinal logistic regression was used for overall dental health grade. Models were adjusted for age, sex, and BMI. Reference categories: dental maturity = normal; overall dental health grade = Good; β-thalassemia major status compared with healthy controls.

**Abbreviations:** OR, odds ratio; CI, confidence interval; BMI, body mass index.

**Table S2. Age-Stratified Descriptive Checks by Corrected Age Group**

| **Age group** | **Case-control status** | **n** | **Delayed dental maturity** | **Good overall dental health grade** | **Fair overall dental health grade** | **Poor overall dental health grade** |
| --- | --- | --- | --- | --- | --- | --- |
| **5–7 years** | β-thalassemia major | 20 | 8/20 (40.0%) | 10/20 (50.0%) | 4/20 (20.0%) | 6/20 (30.0%) |
| **5–7 years** | Healthy controls | 0 | — | — | — | — |
| **8–12 years** | β-thalassemia major | 20 | 15/20 (75.0%) | 7/20 (35.0%) | 4/20 (20.0%) | 9/20 (45.0%) |
| **8–12 years** | Healthy controls | 29 | 9/29 (31.0%) | 20/29 (69.0%) | 7/29 (24.1%) | 2/29 (6.9%) |
| **13–16 years** | β-thalassemia major | 10 | 5/10 (50.0%) | 7/10 (70.0%) | 2/10 (20.0%) | 1/10 (10.0%) |
| **13–16 years** | Healthy controls | 21 | 6/21 (28.6%) | 15/21 (71.4%) | 5/21 (23.8%) | 1/21 (4.8%) |

Age was corrected from 4 to 5 years for one participant based on confirmed data-entry correction. Age groups were used as descriptive checks because some strata contained small or imbalanced case-control counts, limiting the reliability of stratified regression estimates. Overall dental health grade was based on the composite score described in the revised Methods: Good = 0, Fair = 1, Poor ≥2.

**Abbreviations:** β-TM, β-thalassemia major.

**Table S3. Model diagnostics and goodness-of-fit indicators for multivariable regression models**

| **Indicator** | **Delayed dental maturity model** | **Overall dental health grade model** |
| --- | --- | --- |
| Regression model | Binary logistic regression | Ordinal logistic regression |
| Outcome | Delayed dental maturity | Overall dental health grade |
| Adjustment variables | β-TM status, age, sex, BMI | β-TM status, age, sex, BMI |
| Sample size analysed | 100 | 100 |
| Maximum variance inflation factor (VIF) | 1.47 | 1.47 |
| Tolerance range | 0.68–1.00 | 0.68–1.00 |
| Likelihood-ratio test vs null model | χ² = 10.66, df = 4, p = 0.031 | χ² = 10.18, df = 4, p = 0.037 |
| AIC | 136.01 | 193.81 |
| BIC | 149.03 | 209.44 |
| Nagelkerke pseudo-R² | 0.136 | 0.113 |
| McFadden pseudo-R² | 0.078 | 0.053 |
| Residual diagnostics | No influential residual patterns identified | No influential residual patterns identified |
| Influence diagnostics | No observations requiring exclusion | No observations requiring exclusion |
| Proportional-odds assumption | Not applicable | Satisfied (p = 0.925) |

**Note:** Because binary logistic and ordinal logistic regression models were used, conventional adjusted R² values were not applicable. Model fit was therefore evaluated using likelihood-ratio tests, AIC, BIC, and pseudo-R² measures. Variance inflation factors were used to assess multicollinearity, and residual and influence diagnostics were reviewed.

**Abbreviations:** β-TM, β-thalassemia major; VIF, variance inflation factor; AIC, Akaike information criterion; BIC, Bayesian information criterion; R², coefficient of determination.
